# ALG12‐CDG: An unusual patient without intellectual disability and facial dysmorphism, and with a novel variant

**DOI:** 10.1002/mgg3.1304

**Published:** 2020-06-12

**Authors:** María Eugenia de la Morena‐Barrio, María Sabater, Belén de la Morena‐Barrio, Renee L. Ruhaak, Antonia Miñano, José Padilla, Mara Toderici, Vanessa Roldán, Juan R. Gimeno, Vicente Vicente, Javier Corral

**Affiliations:** ^1^ Servicio de Hematología y Oncología Médica Hospital Universitario Morales Meseguer Centro Regional de Hemodonación CIBERER Universidad de Murcia IMIB‐Arrixaca Murcia Spain; ^2^ Servicio de Cardiología Laboratorio de Cardiogenética CIBERCV, Hospital Clínico Universitario Virgen de la Arrixaca‐IMIB Murcia Spain; ^3^ Department of Clinical Chemistry and Laboratory Medicine Leiden University Medical Center Leiden The Netherlands

**Keywords:** ALG12‐CDG, congenital disorder of N‐glycosylation, scoliosis, ventricular septal defect

## Abstract

**Background:**

Congenital disorder of glycosylation (CDG) type I is a group of rare disorders caused by recessive mutations in up to 25 genes that impair the *N*‐glycan precursor formation and its transfer to proteins resulting in hypoglycosylation of multiple proteins. Congenital disorder of glycosylation causes multisystem defects usually with psychomotor delay that is diagnosed in the infancy. We aim to supply further evidences supporting that CDG may be underestimated.

**Methods:**

Antithrombin and factor XI were studied by chromogenic and coagulometric methods. Hypoglycosylation of plasma proteins was evaluated by western blot, HPLC, Q‐TOF, and RP‐LC‐MRM‐MS. Genetic analysis included whole exome, Sanger sequencing, and PCR‐allele specific assay.

**Results:**

We here present an intriguing patient with an exceptional phenotype: 25‐year‐old women with a ventricular septal defect and severe idiopathic scoliosis but no facial dysmorphism, who dances as a professional, and has a University degree. Congenital disorder of glycosylation diagnosis started through the identification of antithrombin deficiency without *SERPINC1* defect and the detection of hypoglycosylated forms. Increased levels of hypoglycosylated forms of F XI (also with significant deficiency) and transferrin were also detected. Whole exome analysis showed a novel homozygous *ALG12* variant c.77T>A, p.(Val26Asp) supporting an ALG12‐CDG diagnosis. It also showed three new variants in *KMT2D*, and a mild, known *ALG6* variant.

**Conclusions:**

This novel ALG12‐CDG patient (the 13th reported) underlines the heterogeneity of this CDG and broadens its phenotypical spectrum, supports that these disorders are underestimated, and suggests that combination of global hypoglycosylation with specific gene defects might determine the clinical manifestations of CDG patients.

## Graphical abstract

We present a case with structural cardiopathy and severe idiopathic scoliosis but not facial dimorphisms (she may be a model!), dances as a professional, and has a University degree. CDG diagnosis was based on antithrombin and FXI deficiency, and increased hypoglycosylated antithrombin, FXI, and transferrin. Exome analysis supported ALG12‐CDG diagnosis. Our model suggests that combination of global hypoglycosylation with specific gene defects might determine the clinical manifestations of CDG patients.

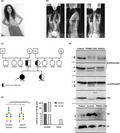

## 1 INTRODUCTION

Congenital disorders of glycosylation (CDG) type I is a group of rare recessive diseases caused by defects in up to 25 different genes involved in the pathway of protein N‐glycosylation (Péanne et al., 2018). Actually, many proteins became hypoglycosylated. The identification of hypoglycosylated forms of transferrin is the gold standard of CDG diagnosis, which is completed by a genetic analysis. The relevance of N‐glycosylation for the folding, intracellular trafficking, and function of many proteins, particularly during development, support multiple mostly severe clinical phenotypes usually comprising also neurological involvement. However, there are patients with very mild phenotypes leading to underdiagnosis of CDG (Péanne et al., 2018). However, there is a considerable clinical heterogeneity, both in terms of severity and organs affected (Péanne et al., 2018). Thus, CDG might be underestimated (Péanne et al., 2018).

## 2 MATERIAL AND METHODS

### 2.1 Ethical compliance

The study was performed in accordance with the International Guideline for Ethical Review of Epidemiological Studies and principles of the Declaration of Helsinki and approved by the ethics committee of Hospital Universitario Reina Sofía de Murcia (8/2013). A written informed consent from patients and healthy subjects was provided.

#### 2.1.1 Patients

The study, in accordance to the ethical standards of the Declaration of Helsinki, was done in a consanguineous Spanish family (parents are cousins). Healthy subjects and PMM2‐CDG patients (Pascreau et al., 2019) were also studied.

#### 2.1.2 Biochemical and functional analysis

Antithrombin activity (anti‐F Xa) and antigen were determined by chromogenic methods and rocket immunoelectrophoresis, respectively. Western blot analysis was used to study plasma proteins (antithrombin, α1‐antitrypsin, F XI, F XII, and prothrombin). F XI was also studied by a clotting assay. Transferrin glycoforms were detected and quantified by HPLC and Q‐TOF (de la Morena‐Barrio et al., 2016). An optimized reversed‐phase liquid chromatography multiple‐reaction monitoring mass spectrometry (RP‐LC‐MRM‐MS) method was used to characterize the glycopeptides of plasma antithrombin (Ruhaak et al., 2018).

#### 2.1.3 Genetic analyses

*SERPINC1* (OMIM: 107300), the gene encoding antithrombin, was evaluated by sequencing and multiplex ligation‐dependent probe amplification, as described (de la Morena‐Barrio et al., 2012). Whole exome sequencing (Ion Torrent) was evaluated using VarAFT 2.6 and Illumina Variant Studio 3.0.12. Sequence variants were checked in public databases (ExAC, gnomAD, 1000 Genomes Project and EVS).

Validation and genotyping of the *ALG12* (OMIM: * 607144) c.77T>A p.(Val26Asp) variant was done by Sanger sequencing and PCR‐allelic specific restriction assay (PCR‐ASRA) with PshaI.

## 3 RESULTS

**FIGURE 1:**
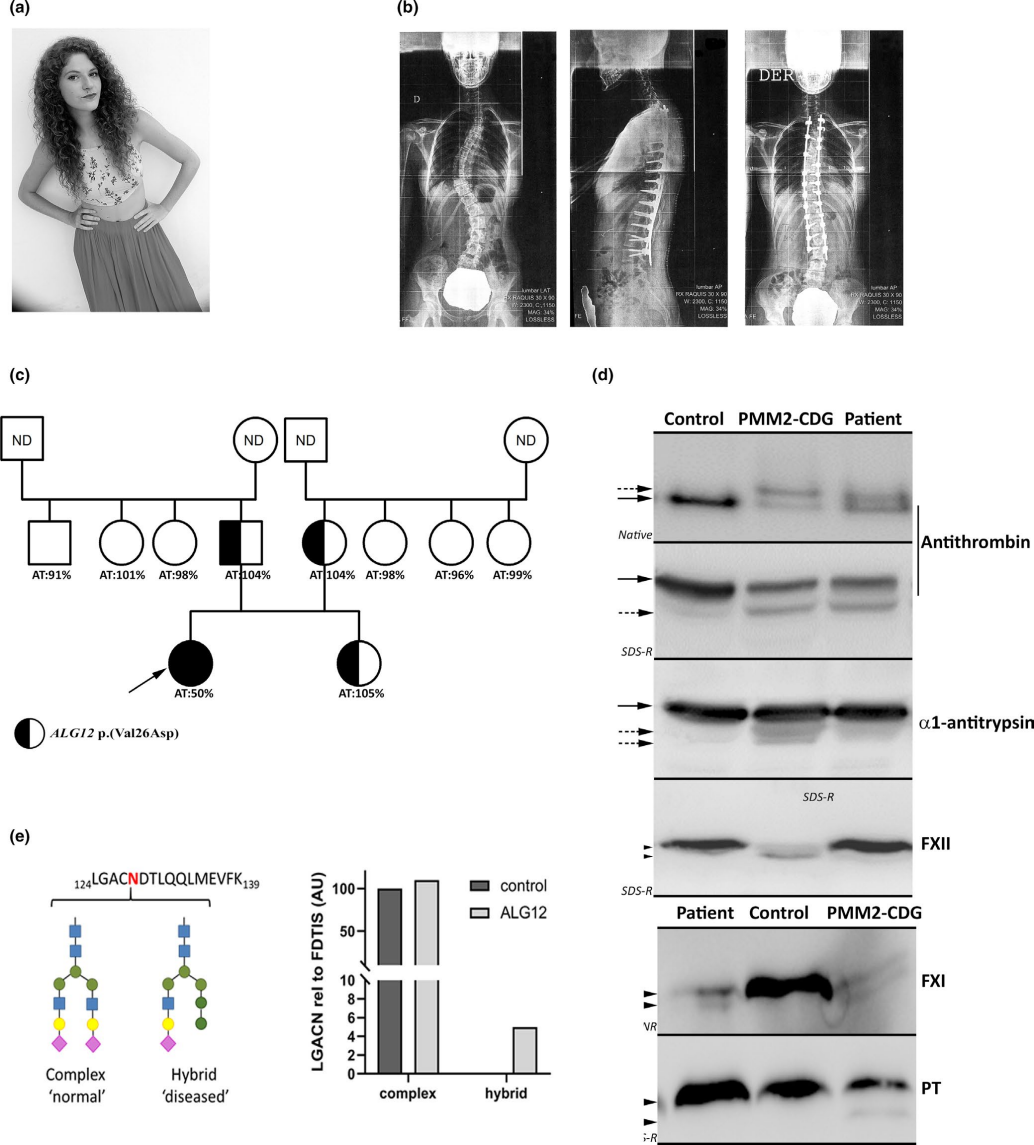
Clinical, biochemical, and genetic characterization of the ALG12‐CDG. (a) Morphological aspect; (b) X‐ray images showing the scoliosis of the before and after the intervention; (c) Family tree. The proband is pointed by an arrow. Anti‐F Xa activity of antithrombin (AT) and the presence of the *ALG12* c.77T>A p.(Val26Asp) variation are shown; (d) Identification of normal (full arrows) and hypoglycosylated forms (dashed arrows) of different proteins (antithrombin; α1‐antitrypsin; Factor XI –FXI; Factor XII –FXII‐, Prothrombin –PT‐) in plasma of the patient, a healthy subject (control), and a PMM2‐CDG patient. The proteins were detected by Western blot after separation using different electrophoretic conditions (Native and denaturing –*SD*‐ under reducing (R) and not reducing –NR‐ conditions). (e) Levels of the complex type glycan and the hybrid‐type glycan at glycopeptide LGACNDTLQQLMEVFK of plasma antithrombin in the ALG12‐CDG patient and a healthy control determined by RP‐LC‐MRM‐MS and calculated relative to the antithrombin quantitative peptide FDTISEK

This 25‐year‐old woman has a university degree, works as a teacher, and dances as well (Figure 1a, supplementary information).

At 4 years of age, a perimembranous ventricular septal defect with misalignment of the interventricular septum and deviation to the left ventricular outflow tract was diagnosed and was successfully corrected. At 11years, she presented idiopathic scoliosis (Figure1b). The scoliosis severely progressed until reaching right thoracic scoliosis T5‐T11 angle of 40° and left thoracic T11‐T1 angle of 35° associated to an important lordosis of 9°. Despite all treatments including the use of different braces (Milwaukee, Cheneoau), she required surgery (posterior arthrodesis from T2 to L3). Moreover, she reported recurrent upper mild respiratory viral infections during infancy.

At 18 years, a thrombophilia screening required by her gynecologist revealed antithrombin type I deficiency (50%). This was at first considered to be spontaneous or an artifact because this is an autosomal dominant disorder and no relatives had antithrombin deficiency (Figure 1c). However, no *SERPINC1* variants were detected, but an antithrombin form with faster electrophoretic mobility was observed by western blot (Figure 1d). RP‐LC‐MRM‐MS analysis of plasma antithrombin showed that the antithrombin‐derived tryptic glycopeptides KANK and SLTFN, which contains two of the N‐glycosylation sites of antithrombin (N167 and N187, respectively) are under‐occupied (ratio related to the non‐glycosylated FDTIS peptide: 0.72 and 0.67, respectively) in the patient compared with healthy controls (*N* = 40, ratio = 1.02 ± 0.19 and 0.85 ± 0.10, respectively). Moreover, the levels of two specific glycopeptides of antithrombin N‐glycosylation site N128 (LGACNDTLQQLMEVFK), one highest abundant complex type glycan and one disease‐specific hybrid‐type glycan, were assessed using targeted mass spectrometry. Levels of the glycopeptides of antithrombin immunoprecipitated from the ALG12‐CDG patient as well as one healthy control were calculated relative to the antithrombin quantitative peptide FDTIS. This study revealed highly similar levels of the complex type glycan in both samples, but the hybrid type glycan was only observed in the ALG12‐CDG patient (Figure 1e). F XI also showed smaller forms in plasma (Figure 1d). The electrophoretic pattern of antithrombin and F XI was similar to that seen in PMM2‐CDG patients (Figure 1d). However, the hypoglycosylation of α1‐antitrypsin, F XII, and prothrombin was mild or undetectable (Figure 1d). Finally, HPLC and Q‐TOF analysis of transferrin glycoforms revealed a CDG type I: asialo‐transferrin: 2.2% (normal range: 0%–1.5%) and disialo‐transferrin 13.8% (normal range: 1%–3%); and a‐ and mono‐glycotransferrin with pathological ratios (a‐/di‐: 0.00779 and mono‐/di‐: 0.27404; CDG‐I ranges: 0.00066–0.45300 and 0.03620–1.06000, respectively). A deficiency of F XI was detected by functional methods (F XI:C = 59%) and Western‐blot (Figure 1d). Importantly, antithrombin and F XI deficiency, and hypoglycosylated forms of transferrin did not change in more than 50 samples collected during 7 years. Other parameters evaluated in the patient, glucose, urea, creatinine, total proteins, albumin, bilirubin, triglycerides, immunoglobulins (IgG; IgA and IgM), cholesterol (HDL and LDL), transaminases, hematocrit, and renal function, all were normal. Only she consistently showed low iron (27 µg/dl), ferritin (7 ng/ml), and saturation index of transferrin (6.3%), with normal folate, transferring, B12 vitamin, and hemoglobin levels.

Whole exome sequencing identified no pathogenic variants in *SERPINC1* or *F11*, but 90 variants were detected in 40 genes involved in CDG. After selecting those implicated in CDG type I, and excluding those located in introns without any role in splicing according to Human Splicing Finder predictions, six potential candidate variants detected (Table S1). Only a homozygous missense variant in *ALG12* [c.77T>A; p.(Val26Asp)], confirmed by Sanger sequencing and by PCR‐ASRA, fulfilled the requirements of a recessive and rare disease. This variant, with very low MAF (not described in EXAC and 0.000008 in TOPMED; rs1208963988), was classified as damaging, or possible damaging by six in silico predictors (Table S1) and is not reported in the *ALG12* mutation database (http://www.hgmd.cf.ac.uk/ac/gene.php?gene=ALG12). Her parents and sister were heterozygous carriers and did not present hypoglycosylation nor antithrombin or F XI deficiency (Figure 1c).

Finally, the search for variants in genes involved in the clinical signs of the patient revealed a known heterozygous *ALG6* variant (p.Ser304Phe) and three new heterozygous variants in *KMT2D. KMT2D* encodes a lysine methyltransferase 2D involved in the Kabuki syndrome, a multisystem autosomal dominant disorder associated to structural cardiopathy and skeletal malformations (Digilio, Marino, Toscano, Giannotti, & Dallapiccola, 2001): ENST00000301067.7: c.10673A>G ENSP00000301067.7: p.(Glu3558Gly). MAF: 0; Polyphen: 0.968; Grantham: 98; ENST00000301067.7: c.3773G>A ENSP00000301067.7: p.(Arg1258Gln). MAF:0; Polyphen: 0.895; Grantham: 43; ENST00000301067.7: c.2527T>C ENSP00000301067.7: p.(Ser843Pro). MAF: 0; Grantham: 74.

## 4 DISCUSSION

CDG is a broad group of hereditary, mostly multisystem disorders, usually diagnosed in the infancy. Thus, nobody might suspect a CDG in our patient. Actually, she was treated by cardiologists and traumatologist without suspicion of an underlying disease. The identification of antithrombin deficiency without *SERPINC1* defects and hypoglycosylation was the first clue of a CDG. Further evidences of a CDG include validation of hypoglycosylation in different proteins (antithrombin, F XI, and transferrin) by different methodological approaches (western blot, HPLC, Q‐TOF, and RP‐LC‐MRM‐MS). Whole exome sequencing leads to the diagnosis of ALG12‐CDG (CDG‐Ig). The *ALG12* encodes Dol‐P‐Man: Man7GlcNAc2‐PP‐Dol‐ mannosyltransferase (or mannosyltransferase 8) that catalyzes the addition of the eighth mannose residue onto the growing lipid‐linked oligosaccharide in the ER. Recently, this enzyme has also been associated to the first steps of maturation of the *N*‐glycan once attached to the protein (Golgi). Thus *ALG12* defects generated under‐occupancy of protein glycosylation sites and potentially aberrant high‐mannose and hybrid‐type structures (Figure S1) (Sturiale et al., 2019), which was supported by the RP‐LC‐MRM‐MS analysis of plasma antithrombin in our patient.

Twelve unrelated ALG12‐CDG patients had been reported, showing 14 unique pathogenic variants (Chantret et al., 2002; Di Rocco et al., 2005; Eklund et al., 2005; Grubenmann et al., 2002; Kranz et al., 2007; Murali et al., 2014; Sturiale et al., 2019; Tahata, Gunderson, Lanpher, & Morava, 2019; Thiel et al., 2002; Zdebska et al., 2003), the first one reported in 2002 (Chantret et al., 2002). Most were diagnosed in infancy or early childhood, and two in adulthood (30 and 44 years). Their phenotypes mainly comprised intellectual disability, growth deficiency, facial dysmorphism with short philtrum and thin upper lips, seizures or stroke‐like episodes, IgG deficiency (usually associated with recurrent infections), and antithrombin deficiency. The mildest phenotype was seen in two adult brothers: only mild/moderate intellectual disability, behavior disturbance, decreased antithrombin and, in one, facial dysmorphism. The present patient also presents a very mild phenotype consisting of scoliosis, a ventricular septal defect, recurrent infections, and decreased antithrombin and F XI activities. There is thus an important heterogeneity in the degree of severity and the phenotypic spectrum of this CDG. This phenomenon is not exceptional in CDG. In PMM2‐CDG, the phenotypes range from neonatal mortality to a near‐normal adulthood (Grunewald, 2009). Other genetic factors probably play a role. In this context, we wonder what the contribution, if any, may be of the coexisting *KMT2D* variants and of the *ALG6* variant. It should be noted that scoliosis, ventricular septal defect, and joint laxity (she is a dancer!) also belong to the Kabuki syndrome spectrum. In conclusion, the present patient shows the mildest ALG12‐CDG presentation reported up to now, although further experimental studies, which might be done by specialized laboratories in ALG12, are required to demonstrate the pathogenic effects of this genetic variant and the associated mechanism.

## CONFLICT OF INTEREST

The authors declare no competing interests.

## AUTHOR CONTRIBUTIONS

MEM‐B, MS, and BM‐B performed research, analyzed data, and wrote and reviewed the paper. AM, JP, and LRH performed genetic and biochemical analysis and analyzed the data. VR performed hemostatic analysis and reviewed the paper. JRG, VV, and JC designed the research, analyzed the data, and wrote and reviewed the paper.

## CONSENT PUBLICATION

The patient gave her consent for information about herself including images and videos to publish in this journal.

## Supporting information

Fig S1

Table S1

Video S1

## Data Availability

All data generated or analyzed during this study are included in this published article [and its supplementary information files].

## References

[mgg31304-bib-0001] Chantret, I. , Dupré, T. , Delenda, C. , Bucher, S. , Dancourt, J. , Barnier, A. , … Moore, S. E. (2002). Congenital disorders of glycosylation type Ig is defined by a deficiency in dolichyl‐P‐mannose:Man‐7‐GlcNAc2‐PP‐dolichyl mannosyltransferase. Journal of Biological Chemistry, 277(28), 25815–25822. 11983712 10.1074/jbc.M203285200

[mgg31304-bib-0002] de la Morena‐Barrio, M. E. , Antón, A. I. , Martínez‐Martínez, I. , Padilla, J. , Miñano, A. , Navarro‐Fernández, J. , … Corral, J. (2012). Regulatory regions of SERPINC1 gene: Identification of the first mutation associated with antithrombin deficiency. Thrombosis and Haemostasis, 107(03), 430–437. 10.1160/TH11-10-0701 22234719

[mgg31304-bib-0003] de la Morena‐Barrio, M. E. , Martínez‐Martínez, I. , de Cos, C. , Wypasek, E. , Roldán, V. , Undas, A. , … Vicente, V. (2016). Hypoglycosylation is a common finding in antithrombin deficiency in the absence of a SERPINC1 gene defect. Journal of Thrombosis and Haemostasis, 14(8), 1549–1560. 10.1111/jth.13372 27214821

[mgg31304-bib-0004] Di Rocco, M. , Hennet, T. , Grubenmann, C. E. , Pagliardini, S. , Allegri, A. E. , Frank, C. G. , … Jaeken, J. (2005). Congenital disorder of glycosylation (CDG) Ig: Report on a patient and review of the literature Journal of Inherited Metabolic Disease, 28(6), 1162–1164. 10.1007/s10545-005-0137-3 16435218

[mgg31304-bib-0005] Digilio, M. C. , Marino, B. , Toscano, A. , Giannotti, A. , & Dallapiccola, B. (2001). Congenital heart defects in Kabuki syndrome. American Journal of Medical Genetics, 100(4), 269–274. 10.1002/ajmg.1265 11343317

[mgg31304-bib-0006] Eklund, E. A. , Newell, J. W. , Sun, L. , Seo, N.‐S. , Alper, G. , Willert, J. , & Freeze, H. H. (2005). Molecular and clinical description of the first US patients with congenital disorder of glycosylation Ig. Molecular Genetics and Metabolism, 84(1), 25–31. 10.1016/j.ymgme.2004.09.014 15639192

[mgg31304-bib-0007] Grubenmann, C. E. , Frank, C. G. , Kjaergaard, S. , Berger, E. G. , Aebi, M. , & Hennet, T. (2002). ALG12 mannosyltransferase defect in congenital disorder of glycosylation type lg. Human Molecular Genetics, 11(19), 2331–2339. 10.1093/hmg/11.19.2331 12217961

[mgg31304-bib-0008] Grunewald, S. (2009). The clinical spectrum of phosphomannomutase 2 deficiency (CDG‐Ia). Biochimica Et Biophysica Acta (BBA) ‐ Molecular Basis of Disease, 1792(9), 827–834. 10.1016/j.bbadis.2009.01.003 19272306

[mgg31304-bib-0009] Kranz, C. , Basinger, A. A. , Güçsavaş‐Calikoğlu, M. , Sun, L. , Powell, C. M. , Henderson, F. W. , … Freeze, H. H. (2007). Expanding spectrum of congenital disorder of glycosylation Ig (CDG‐Ig): Sibs with a unique skeletal dysplasia, hypogammaglobulinemia, cardiomyopathy, genital malformations, and early lethality. American Journal of Medical Genetics. Part A, 143A(12), 1371–1378. 10.1002/ajmg.a.31791 17506107

[mgg31304-bib-0010] Murali, C. , Lu, J. T. , Jain, M. , Liu, D. S. , Lachman, R. , Gibbs, R. A. , … Campeau, P. M. (2014). Diagnosis of ALG12‐CDG by exome sequencing in a case of severe skeletal dysplasia. Molecular Genetics and Metabolism Reports, 1, 213–219. 10.1016/j.ymgmr.2014.04.004 25019053 PMC4088274

[mgg31304-bib-0011] Pascreau, T. , Morena‐Barrio, M. E. , Lasne, D. , Serrano, M. , Bianchini, E. , Kossorotoff, M. , … Borgel, D. (2019). Elevated thrombin generation in patients with congenital disorder of glycosylation and combined coagulation factor deficiencies. Journal of Thrombosis and Haemostasis, 17(11), 1798–1807. 10.1111/jth.14559 31271700

[mgg31304-bib-0012] Péanne, R. , de Lonlay, P. , Foulquier, F. , Kornak, U. , Lefeber, D. J. , Morava, E. , … Jaeken, J. (2018). Congenital disorders of glycosylation (CDG): Quo vadis? European Journal of Medical Genetics, 61(11), 643–663. 10.1016/j.ejmg.2017.10.012 29079546

[mgg31304-bib-0013] Ruhaak, L. R. , Romijn, F. P. H. T. M. , Smit, N. P. M. , van der Laarse, A. , Pieterse, M. M. , de Maat, M. P. M. , … Cobbaert, C. M. (2018). Detecting molecular forms of antithrombin by LC‐MRM‐MS: Defining the measurands. Clinical Chemistry and Laboratory Medicine (CCLM), 56(10), 1704–1714. 10.1515/cclm-2017-1111 29708875

[mgg31304-bib-0014] Sturiale, L. , Bianca, S. , Garozzo, D. , Terracciano, A. , Agolini, E. , Messina, A. , … Barone, R. (2019). ALG12‐CDG: Novel glycophenotype insights endorse the molecular defect. Glycoconjugate Journal, 36(6), 461–472. 10.1007/s10719-019-09890-2 31529350

[mgg31304-bib-0015] Tahata, S. , Gunderson, L. , Lanpher, B. , & Morava, E. (2019). Complex phenotypes in ALG12‐congenital disorder of glycosylation (ALG12‐CDG): Case series and review of the literature. Molecular Genetics and Metabolism, 128(4), 409–414. 10.1016/j.ymgme.2019.08.007 31481313

[mgg31304-bib-0016] Thiel, C. , Schwarz, M. , Hasilik, M. , Grieben, U. , Hanefeld, F. , Lehle, L. , … Körner, C. (2002). Deficiency of dolichyl‐P‐Man:Man7GlcNAc2‐PP‐dolichyl mannosyltransferase causes congenital disorder of glycosylation type Ig. Biochemical Journal, 367(1), 195–201. 10.1042/bj20020794 12093361 PMC1222867

[mgg31304-bib-0017] Zdebska, E. , Bader‐Meunier, B. , Schischmanoff, P.‐O. , Dupré, T. , Seta, N. , Tchernia, G. , … Delaunay, J. (2003). Abnormal glycosylation of red cell membrane band 3 in the congenital disorder of glycosylation Ig. Pediatric Research, 54(2), 224–229. 10.1203/01.PDR.0000072327.55955.F7 12736397

