# Supplementary figures and images for: ALG12‐CDG: An unusual patient without intellectual disability and facial dysmorphism, and with a novel variant
Source: Mol Genet Genomic Med. 2020 Jun 12;8(8):e1304. doi: 10.1002/mgg3.1304 (PMC7434597; doi:10.1002/mgg3.1304)

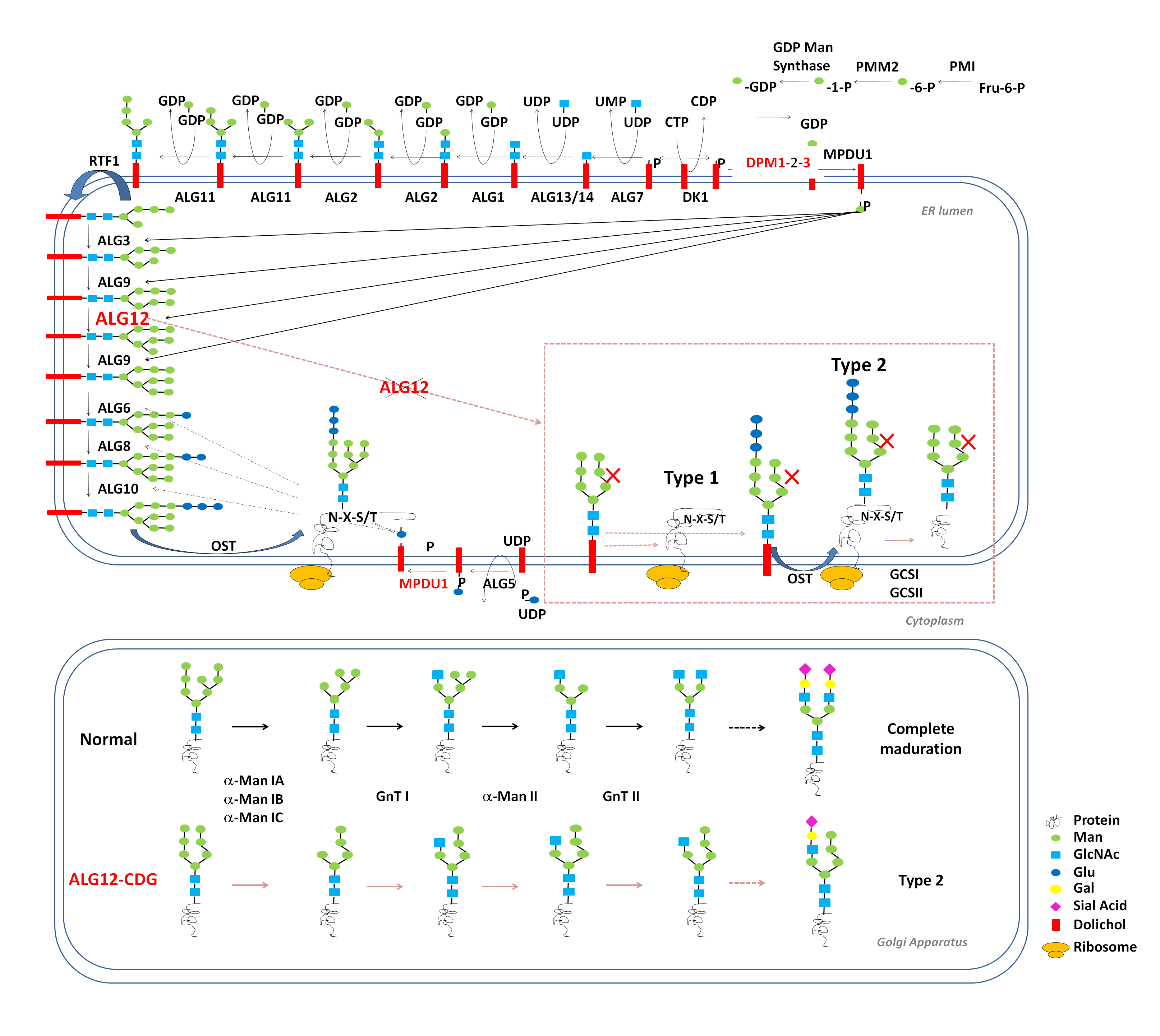

Supplement: Supplementary file 1 — Fig S1 [file MGG3-8-e1304-s001.tif]
